# Reassessment Case: Acute Pulmonary Edema in a Boarding Patient

**DOI:** 10.21980/J8.52353

**Published:** 2025-12-31

**Authors:** Tina Chen, David Fernandez, Amrita Vempati, Kelly Roszczynialski, Stephanie Cohen, Charles Lei, Hillary Moss, Tiffany Moadel, Stephanie Stapleton, Lars Beattie

**Affiliations:** 1 St Louis University, Department of Emergency Medicine, St Louis, MO; 2 Mount Sinai Hospital, Department of Emergency Medicine, Brooklyn, NY; 3 Creighton School of Medicine Phoenix, Department of Emergency Medicine, Phoenix, AZ; 4 Stanford University, Department of Emergency Medicine, Palo Alto, CA; 5 University of Central Florida, Department of Emergency Medicine, Orlando, FL; 6 Hennepin County Medical Center, Department of Emergency Medicine, Minneapolis, MN; 7 Montefiore Medical Center, Department of Emergency Medicine, Bronx, NY; 8 Zucker School of Medicine at Hofstra/Northwell, Department of Emergency Medicine, Hempstead, NY; 9 Boston University/Boston Medical Center, Department of Emergency Medicine, Boston, MA; 10 University of Florida, Department of Emergency Medicine, Gainesville, FL

## Abstract

**Audience:**

The target audience for this communication case is senior residents and junior faculty preparing for the American Board of Emergency Medicine (ABEM) Certifying Exam. Secondary audiences include junior emergency medicine (EM) residents.

**Introduction:**

Rapid identification of clinical changes, reassessment of previous diagnoses, and appropriate adjustment of interventions are critical skills in EM. This case highlights the skills needed to recognize and manage a change in condition with acute pulmonary edema, a life-threatening condition that requires prompt intervention.

**Educational Objectives:**

By the end of the case, the learner should will be able to: 1) demonstrate competency with the new ABEM Certifying Exam Reassessment case format, 2) demonstrate the ability to evaluate new information and integrate it into an existing care plan, 3) recognize signs and symptoms of pulmonary edema, 4) review possible etiologies of acute respiratory distress and the evaluation/work up to differentiate and diagnose those causes, and 5) manage pulmonary edema including implementing afterload reduction, positive pressure ventilation, and diuresis.

**Educational Method:**

This is a standardized patient scenario, in alignment with the expected reassessment case format of the ABEM Certifying Exam. This educational modality is advantageous for assessing the learner’s ability to acquire history and physical examination data in a clinical environment, as well as to communicate with a patient using clear, understandable, and appropriate language.

**Research Methods:**

This case was iteratively evaluated using facilitator and learner surveys at three sites: an academic EM residency program, the 2025 Society for Academic Emergency Medicine Annual Meeting, and a second academic EM residency program. Feedback at each site informed further refinements. A total of 11 senior resident learners and four facilitators tested the case, providing feedback on its quality and usefulness.

**Results:**

Learners and facilitators found the case well-written and effective. All 11 learners felt that the case was helpful practice for the ABEM Certifying Examination. Additionally, all facilitators felt the case materials were easy to use and would use the case again in the future.

**Discussion:**

The case was well-received by both learners and facilitators and appears to be a good preparatory tool for the Reassessment case format of the ABEM Certifying Exam.

**Topics:**

Pulmonary edema, sign-out, reassessment, Certifying Exam.

## USER GUIDE

**Table:** 

List of Resources:	
Abstract	77
User Guide	79
For Examiner Only	81
Certifying Exam Assessment	90
Stimulus	92
Debriefing and Evaluation Pearls	102

**Learner Audience:**

This case is intended for junior and senior residents and junior faculty.

**Time Required for Implementation:**

Case: 20 minutes - 10 minutes for learner to review the door note, 10 minutes for the case

Debriefing: 10 to 20 minutes

**Recommended number of learners per instructor:**

This scenario was designed for one learner per instructor for individual preparation for American Board of Emergency Medicine (ABEM) Certifying Exam. However, in formative contexts, it may be reasonable to have additional residents in an observational role only, or to use this scenario with a junior resident.

**Topics:**

Pulmonary edema, sign-out, reassessment, Certifying Exam.

**Objectives:**

By the end of this practice reassessment certifying exam case, the learner should:

1. Demonstrate competency with the new ABEM Certifying Exam Reassessment case format.
2. Demonstrate the ability to evaluate new information and integrate it into an existing care plan.
3. Recognize signs and symptoms of pulmonary edema.
4. Review possible etiologies of acute respiratory distress and the evaluation/work up to differentiate and diagnose those causes.
5. Manage pulmonary edema including implementing afterload reduction, positive pressure ventilation, and diuresis.

### Linked objectives, methods and results

These objectives were selected to align with general scoring criteria of the ABEM Certifying Exam Reassessment case type (Table 1).^1^ During the scenario, the leader would ideally perform a reassessment of a patient with new onset shortness of breath and increasing respiratory distress (Case Objective 2), diagnosing pulmonary edema based on the patient’s risk factors, vital signs, physical examination, and bedside testing, such as point-of-care ultrasound (POCUS) and portable chest X-ray (Case Objectives 3, 4). Testing should include evidence of consideration of other etiologies (Case Objective 4); for example, an EKG should be ordered to evaluate for acute coronary syndrome as a potential etiology of shortness of breath. The learner should initiate treatment and ensure that appropriate hospital personnel and resources are allocated for treatment delivery (Case Objective 5). Throughout the scenario, they should lead the patient through the stages of the clinical encounter in alignment with expectations of the ABEM Certifying Exam Reassessment case format (Case Objective 1).

### Recommended pre-reading for instructor

- Certifying Exam Sample Case: Reassessment. 2024. Accessed December 9, 2025. This video was released by ABEM as an example of the Reassessment format. We highly recommend that instructors watch this video prior to facilitating this case.
https://www.youtube.com/watch?v=iHMMtBzvGTY
- Swaminathan A. Acute Pulmonary Edema. Core EM. Accessed December 8, 2025.
https://coreem.net/core/ape/
- Hayes, BD. High-Dose Nitroglycerin for Sympathetic Crashing Acute Pulmonary Edema. ALiEM. July 10, 2021. Accessed December 8, 2025.
https://www.aliem.com/high-dose-nitroglycerin-sympathetic-crashing-acute-pulmonary-edema/

### Results and tips for successful implementation

The case was tested on a total of 11 senior resident learners and four EM faculty facilitators, using an iterative case trialing process at multiple sites with a convenience sample of EM residents. Both learners and facilitators provided experience feedback on the quality of case elements via anonymous surveys using Likert scale items and free text comments, based on the Simulation Scenario Evaluation Tool (SSET).^3^ Likert scale items were evaluated on a range of 1 to 5, where 1 = strongly disagree, 2 = disagree, 3 = neutral, 4 = agree, and 5 = strongly agree. All data were collected using Qualtrics (https://www.qualtrics.com) and analyzed using Excel (Microsoft, Redmond, WA). The Boston Medical University Institutional Review Board reviewed the project and deemed it exempt.

During the first round of trialing, a simulation-trained EM faculty member at an academic EM residency program tested the case with two EM senior resident learners. A second round of case trialing was performed at the 2025 Society for Academic Emergency Medicine Annual Meeting in Philadelphia, PA, where two facilitators and two residents completed modified usability surveys. A final round of case trials was performed at a second academic EM residency program, where one facilitator and seven residents completed the same modified usability surveys. This resulted in SSET ratings from a total of four facilitators and 11 senior resident learners. At each stage, the case was refined based on survey feedback.

The SSET data showed favorable perceptions among both learners and facilitators regarding the quality and usability of the case materials. Among the 11 senior resident learners, all indicated agreement or strong agreement that the written materials were clear, and the case was helpful practice for the ABEM Certifying Exam. Ten out of 11 learners agreed or strongly agreed that the verbal instructions were clear, though one learner felt that it was confusing asking the Standardized Patient (SP) for stimuli. Since this appears to be the current practice of the Certifying Exam, the **Standardized Patient Script / Instructions** section was clarified in response to this feedback.

Among the four EM faculty facilitators, all strongly agreed that the case was easy to use, its materials were well integrated, and thought others would feel similarly. All felt confident using the case and would like to use this case for ABEM Certifying Exam practice.

Based on these results, the case appears effective in providing Certifying Exam practice for the Reassessment case format. Though this case can be used in isolation, it may be reasonable to combine it with other cases of different formats for more comprehensive Certifying Exam practice. For the most realistic experience, we recommend deploying the case with one learner. However, depending on the educational context, it may be reasonable for multiple learners in small groups to practice together, or for a senior resident to act as a mock facilitator to better understand an examiner’s perspective.

For an authentic Certifying Exam practice experience, this case requires an SP capable of understanding and adhering to requirements around patient portrayal, case timing, and stimuli prompts, as detailed in **Standardized Patient Script / Instructions**. If the facilitator is responsible for SP training, we recommend that the facilitator reserve time for reviewing and practicing the case with the SP, particularly if the case will be used in a mock summative educational context. Alternatively, in settings where resources preclude a dedicated SP, the facilitator can role play the SP or incorporate manikin-based simulation as an alternative.

## Figures and Tables

**Table:** Some suggested conversation scripting is below:

*Learner Actions*	*Suggested Statements or Responses*
Asks open-ended question about current symptoms, eg, “How are you feeling?” or “I heard from the nurse you are feeling short of breath, is that correct?”	“I’ve been feeling more short of breath for the past two to three hours. This is new since I came to the emergency department.”
Asks questions about events leading to admission.	“I’m still having some pain in my right side, but this has improved since they started the antibiotics. I’m not sure why I’m so short of breath.”
Asks questions about interim history	Provide information as suggested below in **Interim History Details**
Asks for a physical examination, eg, “Can I listen to your lungs?” or “Can I perform a physical examination?”	Provide the learner with **Stimulus 1: Current Physical Examination**.
Discusses the need for bedside testing, specifically point-of-care ultrasound, portable chest X-ray, or EKG	Provide the learner with one or more of the following stimuli, depending on learner statement: - **Stimulus 2: Thoracic Point-of-Care Ultrasound** - **Stimulus 3: Portable Chest X-Ray** - **Stimulus 4: EKG**
Discusses testing that is not provided as a stimulus item, such as a CT scan, fingerstick glucose, etc.	Indicate understanding, eg, “Okay doctor, I’m okay with any test you think I should have.”
Uses overly technical language, such as “SCAPE.”	Indicate confusion, eg, “I don’t understand what you mean, doctor.”
Discusses the diagnosis of pulmonary edema	Please see notes below on additional prompts if the learner does not initiate a discussion about the diagnosis or care plan. Otherwise, say: “Thank you for your help, doctor.”
Discusses the treatment plan	An observing examiner will enter the room and ask the learner to describe the resources they’d like for next steps.
At 8 minutes	There will be a prompt from the examiner: “There are 2 minutes remaining in the case.” This can be used to track time. This may be skipped if the learner seems on track to complete the case.
If 8 minutes pass without the learner discussing the diagnosis.	Ask: “What do you think is going on with me, doctor?” If the learner has not discussed the diagnosis or care plan, ask this question first.
If 10 minutes pass without the learner completing the case.	An observing examiner will enter the room and end the case.

**Table:** Interim History Details:

Chief Complaint	Shortness of breath.
Quality/Character Eg, “How short of breath are you?” “Can you describe your shortness of breath?”	You’re short of breath while talking. You’re too short of breath to leave the bed and walk down the hallway.
Onset Eg, “How quickly did you develop this?” “Did your shortness of breath come on gradually or suddenly?”	Gradual.
Duration/Frequency Eg, “How long has this been going on for?” “Has this happened before?”	Worsening over the past two to three hours. You’ve never experienced this type of shortness of breath before.
Location	Hard to describe. Nothing is hurting.
Radiation	Hard to describe.
Intensity Eg, “How severe is the shortness of breath?”	You’re starting to feel “pretty short of breath” and “pretty worried.”
Aggravating Factors Eg, “Does anything make this worse?”	Movement and walking around make it worse. Talking is starting to be difficult too.
Alleviating Factors Eg, “Does anything make this better?”	If you sit upright, you feel better. But you’re starting to have to concentrate more and more on your breathing.
Associated Symptoms	If the learner asks a broad question about associated symptoms, such as, “Do you have any other symptoms?” You can tell them, “I just feel short of breath.” If the learner asks about specific symptoms, you can give them the following information: - You have no new fever. You had fevers earlier when they diagnosed you with your kidney infection, but that improved with Tylenol. - Your right side still hurts a little, but it’s much better after the antibiotics. - You don’t have cough, leg swelling, calf tenderness, chest pain, nausea, sweating/diaphoresis, presyncope/lightheadedness, or syncope/fainting.

**Table:** Medical History Details:

Past Medical History	Depression Heart failure with reduced ejection fraction, last measured left ventricular ejection fraction of 45%. High cholesterol Hypertension Hypothyroidism
Past Surgical History	You had cardiac catheterization five years ago with left anterior descending artery stent placement. Post-catheterization, you were on dual antiplatelet therapy for one year; currently you only take aspirin and no other antiplatelet therapy.
Hospitalizations	You were hospitalized for your heart attack five years ago.
Medications	aspirin 81mg by mouth once a day amlodipine 10 mg by mouth once a day empagliflozin 10 mg by mouth once a day escitalopram 10 mg by mouth once a day hydrochlorothiazide 50 mg by mouth once a day losartan 100 mg by mouth once a day lovastatin 40 mg by mouth once a day metoprolol tartrate 100 mg by mouth twice a day
Allergies	None
Family History	- parents are deceased - older brother has history of hypertension and myocardial infarction at 50 years of age - younger brother has history of hypertension and diabetes
Substance Use	None, including no current alcohol, tobacco use, or illicit drug use. You previously smoked heavily, but you quit five years ago after your heart attack.
Home Environment	You live in a two-bedroom condo by yourself.
Social History	You were previously married but divorced 10 years ago and prefer to be by yourself. You have no children. You were previously employed as a secretary for more than 40 years. You are active in your local Catholic Charities program and enjoy volunteering several times a week. Your health literacy is good, and you have an above-average understanding of your medical history and previous treatments.

**Table:** 

**Candidate Task Sheet**	
**Emergency Department Patient Chart**	
**#1**	**Current Physical Examination**
**#2**	**Thoracic Point-of-Care Ultrasound**
**#3**	**Portable Chest X-Ray**
**#4**	**EKG**

**Table:** Reassessment Candidate Task Sheet

**CASE PARAMETERS**
This is a 10-minute case You will interact with a standardized patient The patient is admitted to the hospital for treatment of sepsis secondary to pyelonephritis. The nurse has asked you to reassess the patient regarding a new complaint of shortness of breath. You will reassess the patient, develop a plan for diagnosis and treatment, and verbalize your plan to the patient.
**TASK STATEMENT**
Your tasks are as follows: Identify the reason for the patient’s worsening condition Communicate your diagnosis and treatment plan to the patient Describe the personnel and other resources you will need for management

**Table:** Emergency Department Patient Chart

**Patient Name**	Jolene Joseph	
**Age**	61 years old	
**Gender Identity**	Female	
**Presenting Complaint**	Right flank pain	
**History of Present Illness**	Ms. Joseph is a 61-year-old female presenting with right-sided flank pain. Pain began approximately 24 hours ago, associated with subjective fever, dysuria, urinary frequency, and suprapubic pain.	
**Past Medical History**	Depression Heart failure with reduced ejection fraction, last measured left ventricular ejection fraction of 45% High cholesterol Hypertension Hypothyroidism Myocardial infarction with stent placement to the left anterior descending artery five years ago	
**Home Medication**	aspirin 81 mg by mouth once a day amlodipine 10 mg by mouth once a day empagliflozin 10 mg by mouth once a day escitalopram 10 mg by mouth once a day hydrochlorothiazide 50 mg by mouth once a day losartan 100 mg by mouth once a day lovastatin 40 mg by mouth once a day metoprolol tartrate 100 mg by mouth twice a day	
**Allergies**	None	
**PHYSICAL EXAM FINDINGS**		
**Vital Signs on ED Arrival**	BP 110/88, P 110, RR 16/min, T101.8°F (38.8°C), SpO2 98% on room air	
**General Appearance**	Well-developed, well-nourished, pleasant	
**Dermatologic**	Normal	
**HEENT**	Normal	
**Neck**	Normal	
**Respiratory**	Normal	
**Cardiac**	Tachycardic, regular rate and rhythm, Stimuli 1 and 2, no murmurs, gallops, or rubs	
**Abdominal**	Soft, mildly tender to the suprapubic area without rebound/guarding, nondistended, +bowel sounds auscultated	
**Back**	Right-sided costovertebral angle tenderness (CVAT)	
**Extremities**	Normal	
**Neurologic**	Normal	
**Psychiatric**	Normal	
**ASSESSMENT AND PLAN**		
Elderly female presenting with R flank pain, examination notable for fever, tachycardia, R CVAT. Suspect sepsis, most likely source pyelonephritis. Plan on lab work, CXR, fluids, antibiotics per sepsis protocol. Likely admission.		
**MEDICATIONS ORDERED**		
Acetaminophen 1,000mg po		
IVF Bolus: 30cc/kg Ringer’s Lactate		
Cefepime 2g IV		
Vancomycin 2g IV		
**RADIOLOGY RESULTS**		
Chest X-Ray: Normal		
**LAB RESULTS**		
Complete Blood Count	Units	Normal Values
White blood cells	^*^13,100	3,200–9,800/mm^3^
Hemoglobin	14	12–16 g/dL (female)
Hematocrit	42	36–47% (female)
Platelets	212,000	150,000–450,000/uL
Basic Metabolic Panel	Units	Normal Values
Sodium	138	136–145 mEq/L
Potassium	4	3.5–5.0 mEq/L
Chloride	104	98–106 mEq/L
CO2	25	23–38 mEq/L
BUN	15	8–20 mg/dL
Creatinine	0.98	0.7–1.3 mg/dL
Glucose	98	70–105 mg/dL
Calcium	9.2	9–10.5 mg/dL
Troponin	Units	Normal Values
Trop (initial)	<0.1	<0.1 ng/mL
Trop (4 hours later)	<0.1	<0.1 ng/mL
Lactate	Units	Normal Values
Lactate (initial)	^*^2.4	<2.0
Lactate (4 hours later)	1.7	<2.0
Venous Blood Gas	Units	Normal Values
pH	7.34	7.35–7.42
pCO_2_	45.2	42–52 mmHg
pO_2_	24.4	35–45 mmHg
Urinalysis	Value	Normal Values
Color	Yellow	Yellow
Clarity	Clear	Clear
pH	6	4.5–8
Specific Gravity	1.02	1.005–1.025
Glucose	None	<130
Ketones	None	None
Nitrites	^*^Positive	Negative
Leukocyte Esterase	^*^Positive	Negative
Bilirubin	Negative	Negative
Urobilirubin	Negative	Negative
Protein	Negative	Negative
Red Blood Cells	None	<3/hpf
White Blood Cells	^*^25–50	<2–5/hpf
Squamous Epithelial Cells	<10	<10/hpf
**ED COURSE / MEDICAL DECISION-MAKING**		
**Repeat Vital Signs**	BP 112/78, P 105, RR 16/min, T99.8° F (37.7° C), SpO2 96% on room air	
Patient reassessed, non-toxic appearing. R flank pain improved with acetaminophen. Labs and workup notable for elevated WBC, elevated lactate (down trending after sepsis fluids), UA +WBC, +LE, and +nitrites; given concurrent R flank pain, suspect pyelonephritis. Requires antibiotics and inpatient monitoring. Discussed with the medicine admission team, will plan for floor admission. Due to high inpatient volumes, anticipate delay to bed availability. Will sign out to the incoming ED team while awaiting formal transition of care to the hospitalist.		

**STIMULUS 1:** Current Physical Examination

PHYSICAL EXAM FINDINGS
Vital Signs: BP 185/98, P 134, RR 26/min, T98° F (36.7° C), SpO2 88% on 3L O2 by nasal cannula Gen: Well-developed, well-nourished, uncomfortable, diaphoretic Dermatologic: Normal HEENT: Normal Neck: Supple, no lymphadenopathy, +jugular venous distension Respiratory: Diffuse rales throughout all lung fields, no wheezing Cardiac: Tachycardic, regular rate and rhythm, Stimuli 1 and 2, no murmurs, gallops, or rubs Abdominal: Normal Back: Right-sided costovertebral angle tenderness Extremities: Normal Neurologic: Normal Psychiatric: Anxious affect

**STIMULUS 2:**
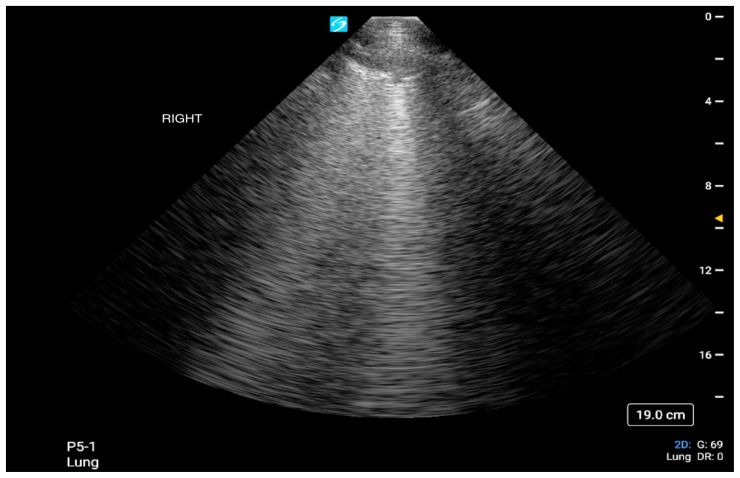
Thoracic Point-of-Care Ultrasound Image Source: Authors’ own image. This finding is bilateral.

**STIMULUS 3:**
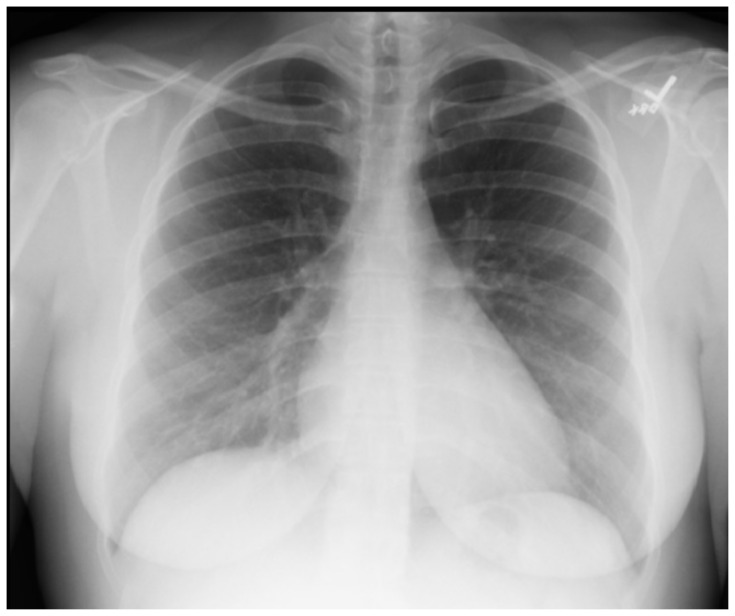
Portable Chest X-Ray Image Source: Authors’ own image.

**STIMULUS 2:**
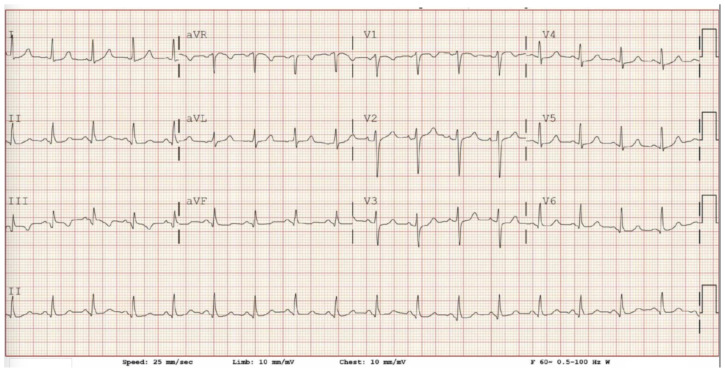
EKG Image Source: Authors’ own image.

**Table 1:** Alignment between ABEM Certifying Examination Reassessment case scoring guidelines^2^ and the developed case objectives:

ABEM CE Reassessment Case Scoring Guideline (as of December 2025)	Case Objective
Obtain additional information after an unexpected change in a case that is in progress Consider systems-based factors	Demonstrate the ability to evaluate new information and integrate it into an existing care plan
Analyze impact of new information	Recognize signs and symptoms of pulmonary edema
Reassess case Describe next steps in patient’s care	Review possible etiologies of acute respiratory distress and the evaluation/work up to differentiate and diagnose those causes
Modify the patient’s care as appropriate Articulate any change in treatment	Manage pulmonary edema including implementing afterload reduction, positive pressure ventilation, and diuresis

**Table 2:** Differential diagnosis of acute dyspnea:

Airway	Pulmonary / Chest Wall	Cardiac	Toxic / Metabolic	Other
Foreign body Angioedema Anaphylaxis Vocal cord dysfunction Trauma	Asthma / COPD exacerbation Pneumonia Pneumothorax Pulmonary embolism Pleural effusion Pulmonary contusion Pulmonary hemorrhage Rib fractures Restrictive lung disease	Acute coronary syndrome / Myocardial infarction Heart failure Pulmonary edema Cardiomyopathy Arrhythmia Valvular heart disease Pericardial effusion / tamponade	Salicylate poisoning Diabetic ketoacidosis Sepsis Other Kussmaul respirations	Anxiety Stroke Neuromuscular disease Anemia

**Table 3:** Key distinguishing features in the presentation, diagnosis, and management of cardiogenic versus non-cardiogenic pulmonary edema:

Category of Pulmonary Edema	“Flash” Pulmonary Edema, or Sympathetic Crashing Acute Pulmonary Edema (SCAPE)^4^	Cardiogenic Pulmonary Edema^5^	Non-Cardiogenic Pulmonary Edema^6^
Pathophysiology	Uniquely dramatic manifestation of acute cardiogenic pulmonary edema Severe systemic hypertension → excessive afterload → fluid shift into lungs	Increased pressure in the left side of the heart → increased pulmonary venous pressure → increased pulmonary capillary pressure → increased alveolar fluid (pulmonary edema)	Direct injury to lung tissue and vasculature
Etiology	Severe systemic hypertension	Coronary artery disease/ myocardial infarction with left ventricular failure Congestive heart failure Cardiomyopathy Mitral or aortic valvular disease Cardiac arrhythmia Right to left shunts	Acute respiratory distress syndrome (ARDS) High altitude pulmonary edema (HAPE) Neurogenic pulmonary edema Opioid overdose Salicylate toxicity Pulmonary embolism Transfusion-related acute lung injury (TRALI)
Onset	Rapid (minutes to hours)	Rapid (minutes to hours) to gradual (days)	Potentially rapid, depending on underlying cause
Manifestations	Rapidly worsening respiratory failure, markedly increased work of breathing, blood-tinged/ pink frothy sputum	If acute/rapid: Marked shortness of breath, systemic congestion may be absent If chronic/gradual: Dyspnea on exertion, orthopnea, paroxysmal nocturnal dyspnea, extremity edema, ascites, weight gain	Rapidly worsening respiratory failure
Key Diagnostics	Patient interview, medical history, collateral sources of information Vital signs, physical examination Bedside cardiac ultrasound or formal echocardiography Lab work, such as electrolytes, troponin, BNP EKG Chest X-ray Pulmonary capillary wedge pressure <18mmHg can rule out cardiogenic pulmonary edema/help rule in non-cardiogenic pulmonary edema^6^		
Key Therapy^4^,^7^	CPAP or BiPAP Nitroglycerin	Volume removal (diuresis/dialysis) If acute/rapid: CPAP or BiPAP	Supportive care Identification and management of underlying cause of event

## FOR EXAMINER ONLY

### Reassessment Case: Acute Pulmonary Edema in a Boarding Patient Summary

**Diagnosis:** Acute pulmonary edema

**Summary of Reassessment:** This case features a 61-year-old female with hypertension and heart failure with mildly reduced ejection fraction who develops pulmonary edema after receiving IV fluids in the treatment of pyelonephritis complicated by sepsis. The learner has 10 minutes to review the initial emergency department (ED) note about this patient, who is boarding in the ED while awaiting an inpatient medicine bed. The learner then has 10 minutes to complete the case.

Ideally, the learner will perform a focused history, request physical examination details (**Stimulus 1: Current Physical Examination**), and ask for bedside diagnostics (**Stimulus 2: Thoracic Point-of-Care Ultrasound**, **Stimulus 3: Portable Chest X-Ray**, and **Stimulus 4: EKG**. These stimuli will be held by the standardized patient and provided to the learner with an appropriate verbal request, as outlined in the **Standardized Patient Script/Instructions** section.

In the ABEM Certifying Examination Reassessment case format, the learner is not required to describe their thought process or differential diagnosis during the scenario. However, the learner’s choice of interview questions and diagnostics may reflect their thought process. Ideally, they will integrate these sources of information and verbalize a diagnosis of pulmonary edema to the standardized patient.

After reaching a diagnosis of pulmonary edema, appropriate choices for stabilizing treatment in this scenario include: 1) positive pressure ventilation, ideally with bilevel positive airway pressure (BiPAP), 2) afterload reduction, for example with nitroglycerin infusion, and 3) diuresis, for example with furosemide or bumetanide. Additionally, the learner will ideally recognize the need for respiratory therapy and stepdown or intensive care unit (ICU) admission.

While this scenario involves interaction with a standardized patient (SP), the examiner should observe the case to evaluate the learner’s actions and complete the **Critical Actions Checklist**. The examiner will also have some key actions to direct case flow, as detailed below.

**Standardized Actor Profile**: Ideally, the SP will be female and approximately 60 years of age. Middle or older age is preferred because this age range makes the diagnosis more plausible and provides a visual clue for the learner. However, depending on the available SPs, the gender and age of the patient can be adjusted. Please see the **Standardized Patient Script/Instructions** section for details about patient portrayal and instructions to the SP regarding case flow.

#### Materials/personnel needed

Examiner
Simulated patient or standardized patient
  Patient gown
  Nasal cannula
  Printouts of stimuli #1–4 in folder
ED stretcher or other patient bed
Vital sign monitor or other virtual representation of vital signs
Clock or timer

**Room Setup:** The room should be set up like an ED room or similar clinical space, with a stretcher, the standardized patient, and a vital sign monitor. There should be a timer or clock prominently displayed. If other items are included in the room, such as a crash cart or ultrasound, the learner must be pre-briefed that they are not needed during the case. There should be a method for an examiner to observe learner actions, eg, on live camera or direct observation through one-way glass.

Initial vital signs displayed on the monitor are:

HR: 134
BP: 185/98
RR: 26
O2Sat: 88% (with the simulated patient on 3L O2 by nasal cannula)

**Play of Case:** The following script can be used to manage the overall case flow and ensure a timely conclusion to the case.

##### Prior to Case Start

- Provide the following instructions: “You are about to encounter an ABEM Certifying Examination case that focuses on reassessment. You will have up to 10 minutes to review a patient chart, and then up to 10 minutes to reassess and initiate management for a patient presenting with a new clinical problem. During the case, you will be expected to perform a focused history and physical examination. You will also be able to request bedside testing, such as point-of-care testing or imaging. You should discuss your diagnosis and plan of care with the patient.”
- Provide the learner with the **Candidate Task Sheet** and **Emergency Department Patient Chart**. At this point, the learner should review these items, which include the patient’s admission note and diagnostic findings.

##### Case Start

- Provide the following instructions: “You can take the patient chart into the examination room with you during the case. Your 10 minutes start now.”

##### At 8 Minute Mark

- Provide the following prompt: “There are 2 minutes remaining in the case.” This prompt is intended to cue the standardized patient to ask about the care plan, if the learner is not yet at this point.
- This prompt may be skipped if the learner has already completed or is imminently about to complete the required case elements.

##### After Learner Describes Care Plan to Patient

- If there is time remaining in the 10-minute scenario, enter the examination room and provide the following prompt: “Doctor, what personnel or hospital resources will you need for your treatment plan?”
- After the learner provides their response, provide the following prompt: “That is the end of your case. Please exit the room and we will proceed to debrief,” or “Please exit the room and proceed to your next examination scenario.”

##### At 10 Minute Mark

- If the learner has run out of time, enter the examination room and provide the following prompt: “The case time has ended. Please exit the room and we will proceed to debrief,” or “Please exit the room and proceed to your next examination scenario.”

**Disposition:** Admit to step down or ICU level of care after reassessment, treatment, stabilization, and communication of clinical status change.

### Reassessment Case: Acute Pulmonary Edema in a Boarding Patient Standardized Patient Script

#### Case Background

You were admitted to this hospital approximately 12 hours ago for pyelonephritis of the right kidney. [Pyelonephritis is an infection of the kidney, usually due to a urinary tract infection that has traveled up the ureter to affect the kidney]. Due to high inpatient volumes, you have been waiting for an inpatient bed in the emergency department (ED). In the past few hours, you began developing shortness of breath, which is likely due to receiving an excessive amount of intravenous fluids.

#### Additional Context

- You have a history of hypertension and heart failure with mildly reduced ejection fraction. You have other medical history as well (see **Medical History Details**), but these are the most relevant ones for this scenario.
- Your hypertension has been difficult to control, and your primary care physician now has you on multiple medications: amlodipine, hydrochlorothiazide, losartan, and metoprolol.
- Your heart failure is very mild and does not typically require diuretics, which are medications that increase urination to decrease total body fluid.
- You were hospitalized five years ago for a heart attack, which required a stent. This was the cause of your heart failure. You have not been hospitalized since. The heart attack presented with chest pain and nausea, but not much shortness of breath. You are not currently on any blood thinners other than a daily aspirin.
- You live alone and don’t have any family who you would like to keep informed of your situation at the moment. You are capable of making your own medical decisions.

##### Your Role in the Case

The learner has been instructed to re-examine you and determine the cause of your new symptoms. They are being evaluated on their ability to reach a correct diagnosis and explain their treatment plan to you. You will be providing information to the learner when they make key requests, as described in the conversation scripting below.

Throughout the case, you should behave as though you are short of breath, which is making you anxious. We suggest that you breathe deeply and more frequently than usual and break up sentences into short sentences due to shortness of breath. You will have a nasal cannula in place that simulates the administration of supplemental oxygen for a low oxygen saturation.

The learner has 10 minutes to review your patient chart prior to entering the room. Once they enter the room and begin interviewing you, they have 10 minutes to complete the case, so details should be provided to them in a straightforward manner. Additionally, given your shortness of breath, you are unable to provide lengthy responses.

#### Ideal Case Flow

The learner will ask you questions about the shortness of breath that began in the past few hours, and you can provide details as suggested in the **Interim History Details** below. They may also ask details of your medical history, which can be provided as suggested in the **Medical History Details** below. They will request a physical examination, allowing you to hand over Stimulus 1. If they request additional bedside testing, such as point-of-care ultrasound, a portable chest X-ray, or EKG, you can hand them the appropriate stimuli (**Stimuli 2, 3, or 4**). Based on this information, they should devise and explain a new treatment plan to you.

## CERTIFYING EXAM ASSESSMENT

*Reassessment Case: Acute Pulmonary Edema in a Boarding Patient*

Learner: _________________________________________

**Obtain additional information after an unexpected change in a case in progress**

□ Obtains focused history
□ Obtains physical examination
  □ Pulmonary Exam
  □ Cardiac Exam
  □ +/− Peripheral Edema

**Analyze impact of new information**

□ Verbalizes pulmonary edema diagnosis

**Modify the patient’s care as appropriate**

□ Verbalizes plan for afterload reduction, eg, nitroglycerin infusion
□ Verbalizes plan for Positive Pressure Ventilation (PPV), eg, BiPAP
□ Verbalizes plan for diuresis, eg, furosemide or bumetanide
□ Verbalized plan is medically reasonable

**Reassess case**

□ Obtains EKG
□ Obtains POCUS
□ Obtains portable chest X-ray

**Describe next steps in patient’s care**

□ Progresses through encounter in an organized manner (history → examination → diagnosis → treatment → closing)
□ Verbalizes encounter stages to patient

**Articulate any change in treatment**

□ Verbalizes diagnosis to patient
□ Verbalizes likely etiology to patient
□ Verbalizes treatment plan to patient
□ Uses clear and understandable language

**Considers system-based factors**

□ Verbalizes need for respiratory therapy
□ Verbalizes need for stepdown or ICU

**Summative and formative comments:**

## DEBRIEFING AND EVALUATION PEARLS

### Reassessment Case: Acute Pulmonary Edema in a Boarding Patient

As part of the 10- to 20-minute debriefing time, facilitators may provide feedback on the following elements:

- The learner’s time management in navigating the case elements. The learner should be capable of completing the case within 10 minutes. Specific items may include the learner’s effectiveness in the following case components:
  - Understanding of the door note/patient chart
  - Logical progression through the clinical encounter, from opening to focused history to physical examination to diagnosis to treatment to closing
  - General understanding of the Certifying Exam case format^1^
- Communication and body language with the standardized patient
  - Use of clear and understandable language
  - Relaxed posture, eye contact, facial expressions
- Clinical reasoning elements
  - Hypothesis generation/differential diagnosis (see Table 2); ideally, learner will be able to describe their initial differential diagnosis and connect physical examination findings from **Stimulus 1: Current Physical Examination** to the correct diagnosis of acute pulmonary edema. Relevant findings include acute respiratory distress, crackles, jugular venous distension, and hypertension.
  - Choice and interpretation of bedside diagnostic tests; ideally, learner would have accessed all available diagnostic **Stimuli**, so any omission of POCUS, EKG, or chest X-ray from the workup can be debriefed and discussed.
  - Management and care plan for pulmonary edema (see Table 3); advanced learners may benefit from a review of cardiogenic versus non-cardiogenic pulmonary edema, as well as acute versus chronic cardiogenic pulmonary edema.
